# Comparison of the clinical effects of lamina replantation and screw fixation after laminectomy in the treatment of intraspinal tumours

**DOI:** 10.1186/s13018-023-04066-z

**Published:** 2023-08-23

**Authors:** Zhen Liu, Ji-Hui Zheng, Na Yuan, Jun Miao

**Affiliations:** 1 Department of Spinal Surgery, Hebei Province Cangzhou Hospital of Integrated Traditional and Western Medicine, 31 Huanghe Road, Cangzhou, 061000 People’s Republic of China; 2 Graduate School, Tianjin Medical University, No. 22 Qixiangtai Road, Heping District, Tianjin, 300070 People’s Republic of China; 3 Department of Spine Surgery, Tianjin Hospital Affiliated to Tianjin University, No. 406 Jiefang South Road, Hexi District, Tianjin, 300210 People’s Republic of China

**Keywords:** Lamina replantation, Tumour resection, Intraspinal tumours, Internalfixation, Pedicle screw

## Abstract

**Introduction:**

Intraspinal tumours are common diseases in neurosurgery and spinal surgery. Due to the fact that most of them are benign tumours, surgical resection is usually effective, and it is also the main treatment for these tumours. To maintain the stability of the spine and to reduce the incidence of kyphosis, pedicle screw fixation is required after traditional laminectomy, but there are many complications. In recent years, tumour resection and laminectomy have become increasingly favoured by clinicians. However, the comparison of the clinical effects of lamina complex replantation and pedicle screw fixation after laminectomy in the treatment of intraspinal tumours is still unknown. This paper systematically compared the two methods from many aspects and discussed their advantages and disadvantages to obtain better clinical guidance.

**Materials and methods:**

In this study, a retrospective analysis was conducted to select 58 patients who underwent posterior approach tumour resection in the spinal surgery department of our hospital from January 2017 to January 2020. Among them, 32 patients underwent tumour resection and laminoplasty, and 26 patients underwent tumour resection and screw internal fixation. The age, sex, body mass index (BMI), smoking status, duration of symptoms, operation time, length of hospital stay, postoperative complications, amount of bleeding and other data were summarized, calculated and compared.

**Results:**

1. The age, sex, BMI, smoking status and symptom duration of the two groups were compared. The abovementioned results were not statistically significant. 2. The operation time, hospital stay, postoperative complications, intraoperative bleeding and adjacent segment degeneration (ASD) were counted and compared between the two groups. There was no significant difference in hospital stay or intraoperative bleeding between the two groups; in addition, the operation time, postoperative complications and incidence of ASD were statistically significant. 3. The visual analog scale (VAS) score, Oswestry Disability Index (ODI) score of thoracic and lumbar spines and Neck Disability Index (NDI) score of cervical spine patients in the two groups were counted, and the preoperative and postoperative data, as well as their changes, were counted and compared between groups and within groups. There was no statistical significance between the two groups; moreover, the postoperative scores were all significantly lower than preoperative in the group. 4. According to the spinal cord function ASIA grade, the preoperative, final follow-up and change values of the two groups were counted, and intragroup and intergroup comparisons were made. There was no significant difference between the two groups; in addition, the scores of the final follow-up were significantly higher than preoperative in the group. 5. The spinal mobility was measured and recorded before the operation and at the final follow-up. There was no significant difference between preoperative and postoperative cervical mobility, and there was no statistical significance observed; furthermore, the range of flexion, extension, rotation and lateral bending of the thoracic and lumbar spines in the screw fixation group was significantly lower than that in the lamina replantation group.

**Conclusions:**

Lamina replantation can be used as splendid methods for the treatment of Intraspinal tumour. Lamina replantation can reduce the operation time, as well as reduce the occurrence of postoperative cerebrospinal fluid leakage, iatrogenic spinal stenosis, posterior soft tissue adhesion and ASD. These complications are reduced in comparison to the other mode of management and better preserve the mobility of the spine.

## Introduction

Intraspinal tumours are common diseases in neurosurgery and spinal surgery. Due to the fact that most of the tumours are benign tumours, surgical resection is usually effective, and it is also the main treatment for this disorder. The basic principle for the treatment of intraspinal tumours is to both completely remove the tumour as much as possible and to protect the stability of the spine as much as possible. Intraspinal canal tumours account for 10–15% of central nervous system tumours [1], and most of them occur in the thoracic vertebrae, lumbar vertebrae and thoracolumbar segments. Extramedullary tumours account for two-thirds of all intraspinal canal tumours [2]. The safe resection of spinal tumours depends on the full exposure of the tumour and its surrounding structures [3], which requires physicians to remove part (or even all of) the lamina when removing the tumour to obtain sufficient surgical vision and operating space. Denis [4] once described the concept of the three-column theory of spines, which furthers our understanding of spine stability. Therefore, it has become a consensus to minimize damage to the original structure of the spine and to restore the stability of the spine. For primary intraspinal tumours, laminectomy is inevitable in order to completely remove the tumour. However, laminectomy destroys the posterior column structure of the vertebral body and affects the biomechanical stability of the spine (to a certain extent). In the long term, it may lead to delayed kyphosis and neurological damage, thus seriously affecting the quality of life of patients [5, 6]. To maintain the stability of the spine and to reduce the incidence of kyphosis, pedicle screw fixation is required after traditional laminectomy. (The fixation of cervical vertebra is mainly lateral mass screw) However, due to the injury and loss of bone and posterior muscle ligament structures, as well as the possibility of epidural fibrosis, cerebrospinal fluid leakage and even iatrogenic spinal stenosis, the range of spinal motion is obviously limited, especially in patients involving multiple segments. This usually leads to the restricted movement of fixed segments and the degeneration of adjacent segments. In addition, scar tissue also has the risk of compressing the spinal cord after surgery [7, 8]. Raimondi first reported of the use of vertebral lamina replantation to clinical treatment, with good surgical effect [9]. Moreover, various laminoplasties have been used to treat patients with intraspinal tumours, with good postoperative effects [10–12]. However, the comparison of the clinical effects of lamina complex replantation and pedicle screw fixation after laminectomy in the treatment of intraspinal tumours is still unknown. Thus, this paper systematically compared the two methods from many aspects and discussed their advantages and disadvantages to obtain better clinical guidance.

## Materials and methods

### General information

**Fig. 1:**
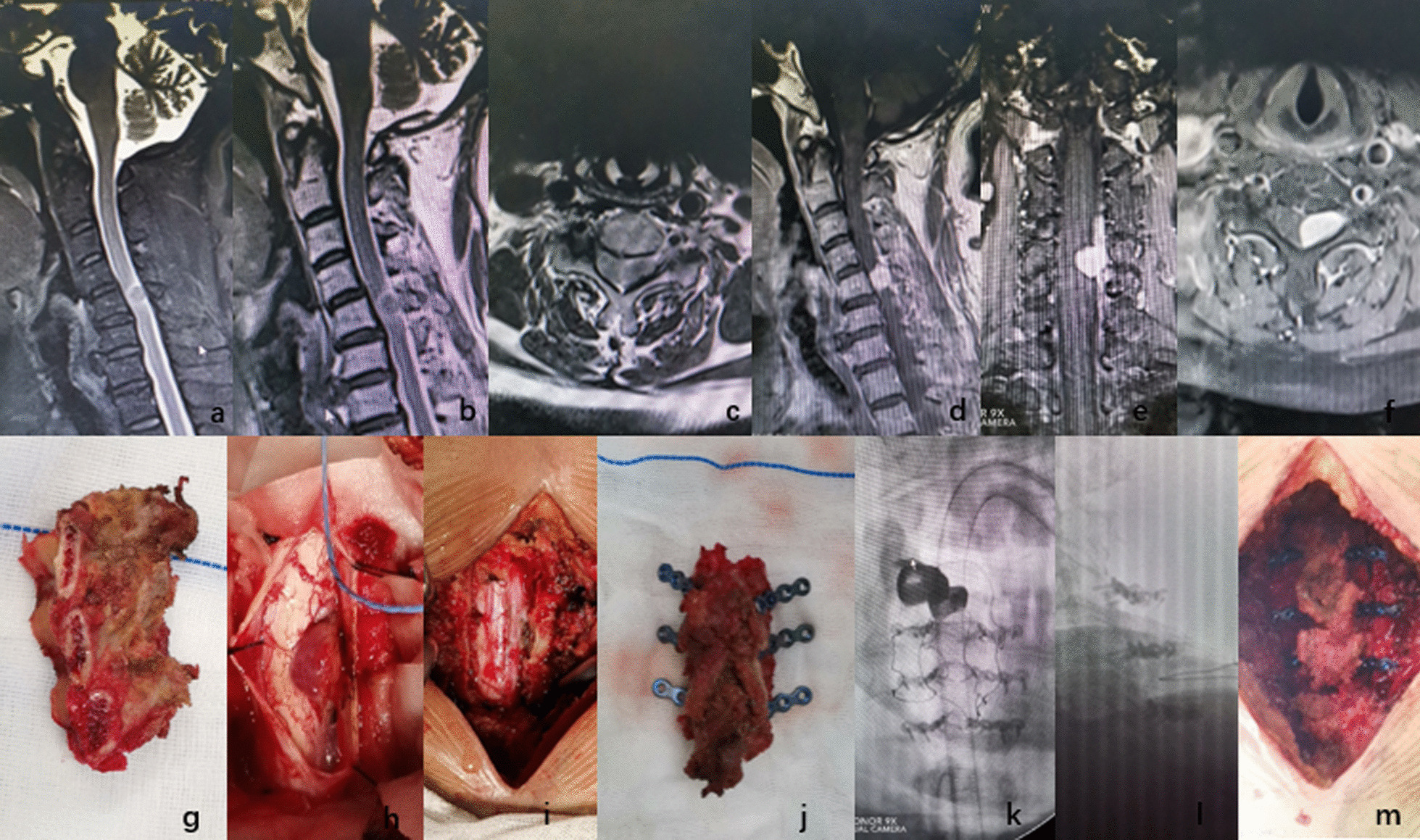
**a** T2WI image of preoperative MRI examination, **b** lipid pressure image of preoperative MRI examination, **c** cross-sectional image of preoperative MRI examination, **d** sagittal position of preoperative enhanced MRI examination, **e** coronal position of preoperative enhanced MRI examination, **f** cross-sectional image of preoperative enhanced MRI examination,** g** completely removed lamina, **h** completely exposed tumour, **i** completely removed tumour, **j** connected lamina of micro compression locking plate, **k** sagittal position X-ray after operation,** l** coronal position X-ray after operation, **m** completely replanted lamina

**Fig. 2:**
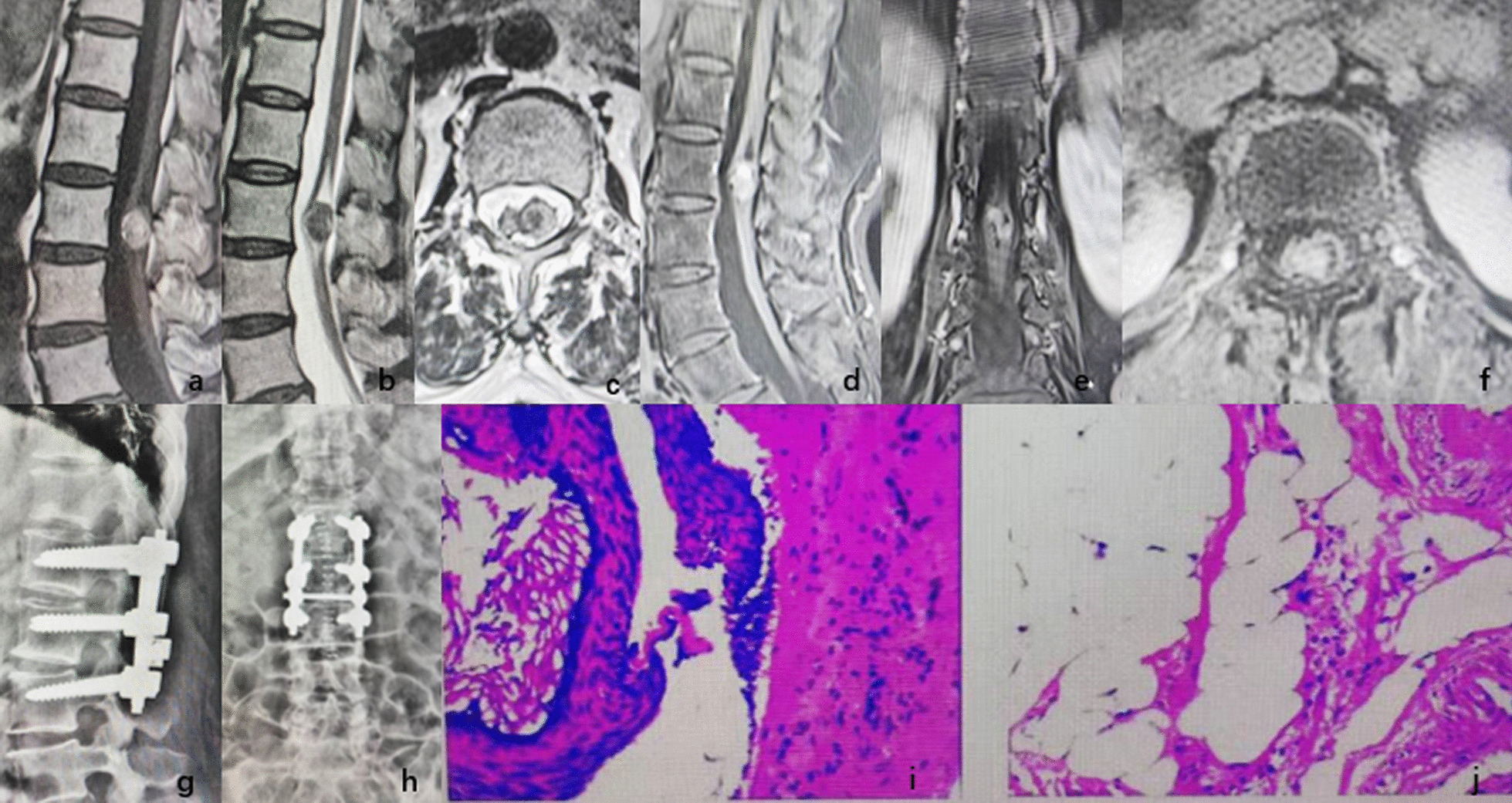
**a** T1WI image of preoperative MRI, **b** T2WI image of preoperative MRI, **c** cross-sectional image of preoperative MRI, **d** sagittal position of preoperative enhanced MRI, **e** coronal position of preoperative enhanced MRI, **f** cross-sectional image of preoperative enhanced MRI, **g** and **h** pedicle screw fixation after tumour resection, **i** and **j**: mature cystic teratoma shown by pathology

The study was approved by the ethical committee of Tianjin Hospital. The requirement for written informed consent was waived by the ethics committee of Tianjin Hospital because of the retrospective nature of the study. This study used a retrospective analysis to select 58 patients who underwent posterior approach tumour resection for intraspinal tumours in the spine surgery department of our hospital from January 2017 to January 2020. The operation was performed by the same surgeon. The main symptoms of the patient were numbness, fatigue, hypoesthesia, chest waist girdle sensation, root pain and other symptoms, which were consistent with the surgical indications. In this 2-year follow-up study, 58 patients were finally included in the study, including 32 patients (24 patients with thoracolumbar vertebrae and 8 patients with cervical vertebrae) who received tumour resection and lamina complex replantation (Fig. 1) and 26 patients (20 patients with thoracolumbar vertebrae and 6 patients with cervical vertebrae) who received tumour resection and pedicle screws (including lateral mass screws) and internal fixation (Fig. 2). The two surgical methods have similar indications for resection of intraspinal tumours, and there is no clear literature on the selection criteria for both surgical methods. Therefore, the two surgical methods were randomly performed among patients. Preoperative routine X-ray films, computed tomography (CT) and magnetic resonance imaging (MRI) were performed to observe the horizontal displacement and angle changes of each segment, and the presence of preoperative spinal instability was excluded. Routine electromyography was performed to exclude neurogenic lesions. After routine scanning, all of the patients underwent enhanced scanning to confirm the scope and size of the tumour and its relationship with the spinal cord, cauda equina and nerve root.

The following inclusion criteria were used: (1) single or adjacent multiple tumours, as well as epidural or extramedullary subdural tumours; (2) the patients were followed up for at least 2 years with complete data; (3) the patient received spinal surgery for the first time; and (4) there was no spinal instability or structural damage.

The exclusion criteria were as follows: (1) patients who were followed up for less than 2 years for various reasons; (2) articular process joint destruction; (3) recurrent intraspinal tumours; and (4) metastatic intraspinal tumours.

### Operation method

#### Resection of the tumour and replantation of the lamina complex

**Fig. 3:**
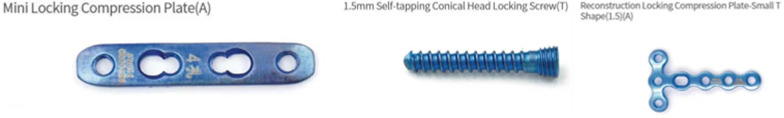
Common locking plates and their screws: mini locking compression plate, self-tapping conical head locking screw, reconstruction locking compression plate-small T shape

All of the patients were under general anaesthesia with endotracheal intubation. After successful anaesthesia, they were prone on the spinal operating table. Head frames were used for the cervical spine and cervical thoracic segment. Mayfield stents were used for the severe patients. According to the fluoroscopic localization of the tumour segment, a posterior median longitudinal incision was made (with the tumour segment as the centre), the skin and subcutaneous tissue were cut, the bilateral muscle tissues were stripped, the integrities of the supraspinous ligament and interspinous ligament were preserved, and the spinous process, lamina and bilateral facet joints were exposed. The exposure range included the tumour segment and one lamina above and below. Moreover, the placement position of the mini plate was designed, and the mini plate was bent at the segment where the lamina was to be cut. The bilateral laminae were cut by an ultrasonic osteotome from approximately 2–3 mm inside of the facet joint. Additionally, the cutting direction involved the ultrasonic osteotome head being inclined to the midline and sagittal plane at approximately 5–10°. The range of bone cutting was approximately 5 mm longer than that of the proximal and distal ends of the tumour. Attention was given to the protection of the interspinous ligament and the supraspinous ligament. In addition, ultrasonic osteotome was used to cut spinous process and lamina. The spinous process lamina ligament complex was completely removed, and the precurved microcompression locking plate was fixed on the spinous process lamina ligament complex for standby. With the aid of a microscope, the dura mater was cut, the tumour was completely removed, and the dura mater was sutured. The spinous process lamina ligament complex was reset and fixed with screws (Fig. 3). Furthermore, the drainage tube was placed, the nuchal ligament or lumbar dorsal fascia was sutured to the supraspinous ligament, and the incision was sutured layer by layer.

#### Tumour resection and screw internal fixation

The preliminary preparation was the same as mentioned above in the prone position, and a posterior median longitudinal incision was made with the tumour segment as the centre. The skin and subcutaneous tissue were sliced. After separating the ligaments, the muscles were stripped, screws were placed on both sides, the vertebral lamina was removed, and the ligamentum flavum was removed to expose the dura mater. Additionally, we completely exposed the tumour, separated and removed the tumour, completely stopped bleeding, carefully checked whether all of the tumours were removed and closely sutured the dura mater. The screw rod system was used for internal fixation, the drainage tube was placed, and the muscle, fascia and skin layers were closely sutured.

### Observation indicators and efficacy evaluation

Patient age, sex, body mass index (BMI), smoking status, symptom duration, operation time, hospital stay, postoperative complications, bleeding volume and other data were summarized, calculated and compared. Adjacent segment degeneration (ASD) was defined as abnormal changes in adjacent motion segments in the fusion area after spinal fusion, including the loss of intervertebral height, intervertebral disc degeneration or protrusion, spondylolisthesis, vertebral instability, osteophyte formation and vertebral compression fracture. According to whether there are clinical symptoms, ASD can be classified into adjacent segment degeneration and adjacent segment disease. Adjacent segment degeneration is defined as imaging degeneration of adjacent segments without clinical symptoms after fusion, whereas adjacent segment disease exhibits new symptoms corresponding to imaging changes of adjacent segments in the fusion area [13, 14]. The ASD was not subdivided in this study. The visual analogue scale (VAS) was used to evaluate the pain of the patients, and the Oswestry Disability Index (ODI) was used to evaluate the function of the thoracic and lumbar spines. Moreover, the Neck Disability Index (NDI) was used to evaluate the function of the cervical spine. Neurological status was assessed by using the ASIA grade. To determine the level of internal fixation and the stability of the spine, the spine was examined via frontal and lateral X-rays at the postoperative outpatient re-examination.

### Range of motion (ROM) measurement

Based on the Guidelines for Range of Motion Indication and Measurement established by the Japanese Orthopedic Association and Japanese Association of Rehabilitation Medicine in 1995, the following ROM were measured using an international standard goniometer (S7025 l; MINATO MEDICAL SCIENCE, Osaka, Japan): the cervical spine (flexion, extension, rotation and lateral bending); thoracic and lumbar spines (flexion, extension, rotation and lateral bending). Each range was measured three times to calculate the mean.

For cervical vertebral mobility, the patient was in a sitting or standing position with his head in the middle and the eyes looking straight ahead. We selected the appropriate axis, fixed the arm and mobile arm for measurements and correspondingly checked the following actions. (1) For flexion, the examiner asked the patient to touch the chest with the chin to estimate the activity of the cervical spine. (2) For extension, the examiner asked the patient to look up as much as possible. (3) For lateral bending, the patient was asked to touch the right shoulder with the right ear and the left ear with the left shoulder. The equal height of the shoulders was focused on in advance. The patient was instructed to not lift the shoulders during the movement. (4) For rotation, the subject was asked to touch the left and right shoulders with the chin but to not lift the shoulders to touch the chin. The thoracic and lumbar spines were detected by a similar method, and the thoracic and lumbar spines ROM was verified.

### Statistical methods

SPSS software was used for the statistical analysis. The measurement data between the two groups are expressed as the mean ± standard deviation. The independent sample t test or Mann‒Whitney U test was applied to compare the relevant results between the groups (according to whether the data conformed to a normal distribution), and the paired t test or nonparametric test was applied to compare the relevant results within groups (according to whether the data conformed to a normal distribution). The counting data were analysed by using the chi square test, and *p* < 0.05 was considered to be statistically significant.

## Results

1. Patient characteristics

The age, sex, BMI, smoking status and symptom duration of the two groups were counted and compared (Table 1). Age (lamina replantation group: 56.91 ± 9.04-years-old; screw fixation after laminectomy group: 60.96 ± 8.16-years-old; *p* = 0.08), sex (lamina replantation group: 17 males and 15 females; screw fixation after laminectomy group: 14 males and 12 females; *p* = 0.96), BMI (lamina replantation group: 24.74 ± 5.53; screw fixation after laminectomy group: 25.37 ± 4.49; *p* = 0.64), smoking status (lamina replantation group: 17 smoking subjects and 15 nonsmoking subjects; screw fixation after laminectomy group: 13 smoking subjects and 13 nonsmoking subjects; *p* = 0.81) and the duration of symptoms (10.31 ± 4.32 days in the lamina replantation group and 12.46 ± 5.73 days in the screw fixation after laminectomy group; *p* = 0.11) were not statistically significant.

**Table 1:** Comparison of patient characteristics between the two groups

	Lamina replantation (*n* = 32)	Screw fixation after laminectomy (*n* = 26)	*p*-value
Age (year)	56.91 ± 9.04	60.96 ± 8.16	0.08
Sex (male/female)	17/15	14/12	0.96
Body mass index (BMI)	24.74 ± 5.53	25.37 ± 4.49	0.64
Current smoker (Y/N)	17/15	13/13	0.81
Duration of symptoms(day)	10.31 ± 4.32	12.46 ± 5.73	0.11

2. Operation-related parameters

The operation time, hospital stay, postoperative complications, intraoperative bleeding and ASD of the two groups were counted and compared (Table 2). There was no significant difference in hospitalization time or intraoperative bleeding between the two groups. Operation time (116.56 ± 30.75 in the lamina replantation group; 133.46 ± 32.24 in another group; *p* < 0.05) and postoperative complications (5 cases in the lamina replantation group; 18 cases in another group; *p* < 0.001) were statistically significant. Postoperative complications were defined as: postoperative cervical fluid leakage, iatrogenic spinal stenosis, posterior soft tissue adhesion. ASD (not observed in the lamina replantation group, and 2 cases observed in another group; *p* < 0.001).

**Table 2:** Comparison of operation-related parameters between the two groups

	Lamina replantation (*n* = 32)	Screw fixation after laminectomy (*n* = 26)	*p*-value
Operation time (minutes)	116.56 ± 30.75	133.46 ± 32.24	< 0.05*
Length of hospitalization(days)	7.5(6 ~ 11)	6.0(5 ~ 10)	0.20
Complications(N)	5	18	< 0.001*
Blood loss(ml)	146.25 ± 31.39	137.35 ± 28.28	0.27
ASD(N)	0	2	< 0.001*

Complications: postoperative cerebrospinal fluid leakage, iatrogenic spinal stenosis, postoperative soft tissue adhesion

* Statistically significant *p*-values were the results after comparison between the two groups

3. VAS and ODI/NDI scores

The VAS score, ODI score (thoracic and lumbar spines) and NDI score (cervical spine) in the two groups were counted; in addition, the preoperative, postoperative and change values were counted, and intergroup and intragroup comparisons were made (Table 3). There was no statistically significant difference between the two groups. Moreover, the postoperative scores of the two groups were significantly lower than 0.001.

**Table 3:** Comparison of VAS and ODI/NDI scores between the two groups

	Lamina replantation (*n* = 32)	Screw fixation after laminectomy (*n* = 26)	*p*-value
VAS			
Preoperative	5.19 ± 2.26	4.73 ± 1.89	0.42
Final follow-up	1(0 ~ 2)	1(1 ~ 2)	0.52
ΔVAS	4.00 ± 2.36	3.46 ± 2.14	0.37
*p*-value	< 0.001*	< 0.001*	
ODI	(*n* = 24)	(*n* = 20)	
Preoperative	66.56 ± 6.14	68.12 ± 6.62	0.36
Final follow-up	15.25 ± 5.25	15.12 ± 3.50	0.91
ΔODI	51.31 ± 7.37	53.00 ± 7.46	0.39
*p*-value	< 0.001*	< 0.001*	
NDI	(*n* = 8)	(*n* = 6)	
Preoperative	23.38 ± 5.32	23.67 ± 3.50	0.91
Final follow-up	12.38 ± 3.11	13.17 ± 2.32	0.61
ΔNDI	11.00 ± 7.17	10.50 ± 4.46	0.88
*p*-value	< 0.001*	< 0.001*	

The thoracic and lumbar spines function was evaluated by ODI scores (24 cases in lamina replantation group and 20 cases in screw fixation after laminectomy group)

The cervical spine function was evaluated by NDI scores (8 cases in Lamina replantation group and 6 cases in screw fixation after laminectomy group)

ΔThe difference between final follow-up and preoperative

* Statistically significant *p*-values were the results after comparison between the two groups

4. ASIA grade

**Fig. 4:**
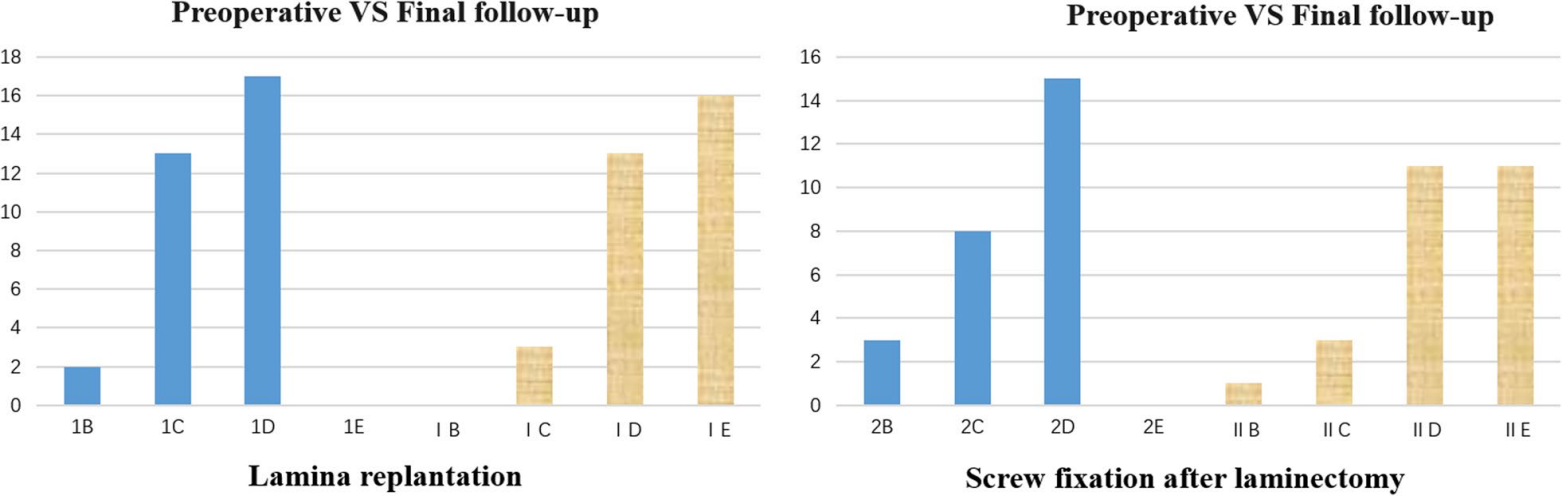
Draw two groups of ASIA grade bar charts according to the data in Table 4

According to the spinal cord function ASIA grade, the preoperative, final follow-up and change values of the two groups were counted. The data were collected by the assignment method and compared within and between the groups (Table 4). No statistically significant difference was found between the two groups. The scores of the final follow-up in the group were higher, and the *p* values were less than 0.001 (with a significant difference being observed) (Fig. 4).

**Table 4:** Comparison of ASIA grade between the two groups

	Lamina replantation (*n* = 32)	Screw fixation after laminectomy (*n* = 26)	*p*-value
ASIA			
Preoperative	3(2 ~ 3)	3(2 ~ 3)	0.90
Final follow-up	3.5(3 ~ 4)	3(3 ~ 4)	0.22
ΔASIA	1(1 ~ 1)	1(0 ~ 1)	0.10
* p*-value	< 0.001*	< 0.001*	

Give ABCDE corresponding values, respectively: A = 0 B = 1 C = 2 D = 3 E = 4

* Statistically significant *p*-values were the results after comparison between the two groups

5. Spinal ROM

The ROM of the spine was measured and recorded before the operation and at the final follow-up. Measurements of the ROM of the thoracic and lumbar spines (Table 5) for patients with thoracic and lumbar spine tumours and measurements of the ROM of the cervical spine (Table 6) for patients with cervical spine tumours were performed. There was no significant difference between preoperative and postoperative cervical mobility. Furthermore, the range of flexion, extension, rotation and lateral bending of the thoracic and lumbar spines in the screw fixation group was significantly lower than that in the lamina replantation group.

**Table 5:** Comparison of thoracic and lumbar spines ROM between the two groups

	Lamina replantation (*n* = 24)	Screw fixation after laminectomy (*n* = 20)	*p*-value
Thoracic and lumbar spines flexion			
Preoperative	42.00(39.00 ~ 44.00)	41.50(37.25 ~ 43.00)	0.46
Final follow-up	42.00(39.25 ~ 43.75)	35.50(34.00 ~ 39.00)	< 0.001*
Thoracic and lumbar spines extension			
Preoperative	26.00(25.00 ~ 27.00)	26.00(24.25 ~ 28.00)	0.45
Final follow-up	25.58 ± 1.91	24.00 ± 2.55	< 0.05*
Thoracic and lumbar spines left rotation			
Preoperative	41.50(39.00 ~ 43.00)	41.00(37.25 ~ 42.75)	0.34
Final follow-up	41.00(40.00 ~ 43.00)	38.50(35.25 ~ 40.00)	< 0.001*
Thoracic and lumbar spines right rotation			
Preoperative	41.50(39.00 ~ 43.00)	41.00(37.00 ~ 42.75)	0.48
Final follow-up	41.00(40.00 ~ 43.00)	38.00(34.50 ~ 40.75)	0.001*
Thoracic and lumbar spines left lateral bending			
Preoperative	36.13 ± 2.03	35.10 ± 2.53	0.14
Final follow-up	36.17 ± 2.06	33.60 ± 2.68	0.001*
Thoracic and lumbar spines right lateral bending			
Preoperative	36.21 ± 2.28	35.15 ± 2.32	0.14
Final follow-up	36.17 ± 2.18	33.60 ± 2.98	< 0.05*

* Statistically significant *p*-values were the results after comparison between the two groups

**Table 6:** Comparison of cervical spine ROM between the two groups

	Lamina replantation (*n* = 8)	Screw fixation after laminectomy (*n* = 6)	*p*-value
Cervical flexion			
Preoperative	49.88 ± 8.81	53.00 ± 9.01	0.53
Final follow-up	49.63 ± 7.03	52.83 ± 8.40	0.45
Cervical extension			
Preoperative	44.88 ± 5.30	46.83 ± 2.99	0.44
Final follow-up	45.38 ± 4.96	46.67 ± 2.50	0.57
Cervical left rotation			
Preoperative	63.25 ± 6.07	64.50 ± 4.81	0.69
Final follow-up	63.75 ± 6.14	64.33 ± 4.72	0.61
Cervical right rotation			
Preoperative	63.00 ± 6.23	64.50 ± 4.85	0.64
Final follow-up	63.00 ± 5.98	64.17 ± 4.71	0.70
Cervical left lateral bending			
Preoperative	45.75 ± 3.01	44.67 ± 2.50	0.49
Final follow-up	45.50 ± 2.73	44.67 ± 1.51	0.52
Cervical right lateral bending			
Preoperative	45.75 ± 3.01	44.50 ± 2.43	0.42
Final follow-up	45.63 ± 2.77	44.67 ± 1.21	0.45

## Discussion

Most intraspinal tumours in adults are benign tumours, with multiple occult diseases, atypical symptoms and progressive aggravation, which can eventually lead to compression of spinal nerve roots and even paralysis in severe cases. Surgical resection is considered to be the only effective treatment. At present, early diagnosis and complete resection are advocated in the clinical setting to relieve the compression of the spinal cord and nerve root in a timely manner. According to the relationship between the spinal cord and the tumour, the surgical approaches are generally divided into posterior approaches, anterior approaches and combined anterior and posterior approaches. However, the exposure of anterior surgery is difficult, and it easily damages blood vessels and nerves. In addition, the incidence of intraspinal tumours in the dorsal spinal cord was reported to be much higher than that in the ventral spinal cord. Therefore, the posterior approach has become a conventional surgical approach [15]. According to the "three-column theory", the posterior column possesses approximately 24–30% of the pressure and approximately 21–54% of the rotational stress [16]. The integrity of the posterior column structure is very important to ensure the stability of the spine. Therefore, the complete removal of intraspinal tumours and the protection of spinal anatomy and function are the basic principles of intraspinal tumour resection [17]. The traditional surgical method is to remove all of the vertebral lamina and spinous processes of the diseased segments. This resection method makes the paravertebral muscles lose their normal bony attachment points, and the posterior column structure loses its function as a tension band. The following series of complications can occur: spinal instability, kyphosis and symptomatic epidural scar formation. Among them, the most common complication is kyphosis, especially in patients with cervical intraspinal tumours [18, 19].

In recent years, spine doctors have paid increasing attention to the stability of the spine. Due to the fact that tumour resection and pedicle screw fixation can fully expose the tumour and create a good space for tumour removal, they also maintain the stability of the spine and reduce the probability of postoperative spinal instability and kyphosis. It has gradually become the mainstream operation for this disorder, especially for patients requiring long segment exposure; however, it still has many problems, such as internal fixation fracture, cerebrospinal fluid leakage, epidural haematoma and soft tissue adhesion. In addition, the internal fixation system also destroys the physiological functions of flexion, extension and rotation of the spine to a certain extent, as well as accelerating the degeneration of adjacent segments of the spine and causing adjacent segment degeneration, including intervertebral disc herniation, osteophyte formation and spinal canal stenosis. Therefore, as a new technology, spinous process and lamina replantation laminoplasty is increasingly being respected as an approach by clinicians [20]. It has the following advantages: it retains the spinous process ligament complex, maintains the posterior tension of the spine and reduces the occurrence of kyphosis; in addition, the dural sac is protected by the bone to reduce scar formation and to prevent iatrogenic spinal stenosis, and the maintenance of the function of the spinal motion segment is in line with the concept of nonfusion surgery [21].

From the results, we observed that the operation time of patients in the lamina replantation group is significantly lower than that in the screw fixation after laminectomy group, which is not unexpected because the process of lamina replantation is much simpler than that of screw implantation, and the incidence of postoperative complications (postoperative cerebrospinal fluid leakage, iatrogenic spinal stenosis and postoperative soft tissue adhesion) in the lamina replantation group was significantly lower than that in the control group, which is also in line with previous literature reports. Due to the fact that the soft tissue in the spinal canal is protected after the replantation of the lamina, the spinal canal is separated from the rear muscle to reduce stimulation and adhesion. Many studies aimed at preventing postoperative epidural adhesion (such as the use of adipose tissue, amniotic membrane, silicone membrane and silicone rubber, as well as the dripping of hormones and anti-inflammatory drugs) have no obvious effects [22–24]. However, we believe that lamina replantation is an effective and safe method to prevent scar adhesion after spinal surgery. Lamina replantation prevents the posterior tissue from protruding into the spinal canal, thus compressing the spinal cord and causing pain, numbness, fatigue and other symptoms of the waist and legs, which then leads to the failure of the operation and the risk of secondary surgery [25, 26]. The in situ replantation of the spinous process and lamina complex makes the dural incision close to the inner surface of the lamina, which can ultimately reduce the risk of cerebrospinal fluid leakage. Our study showed that the incidence of ASD in the screw fixation group was significantly higher than that in the lamina replantation group, which may be due to the loss of spinal mobility after internal fixation, thus resulting in increased stress on adjacent segments and even borderline kyphosis. It is well known that the spine is affected by various load conditions, among which the invariable rule is joint movement. The movement of one segment corresponds to the mutual movement of another segment, which is a necessary prerequisite for understanding the interaction between bones, ligaments and intervertebral discs. The decompression of the spinal cord and nerve roots usually requires the removal of some structures that maintain spinal stability, such as the lamina and facet process, which changes the biomechanics of the adjacent segments. Previous research has shown that the fusion and fixation of the moving segments increases the stress of the adjacent segments at the cephalic side [27]. Smith Robinson et al. conducted finite element analysis after spinal fusion surgery, which also showed that the internal stress of the cephalic segment increased [28]. Furthermore, Matsunaga et al. [29] reported that the shear stress of the adjacent segments increased by 20% at one year after multi-level fusion. Increasing evidence has quantified the effect of adjacent segment shear force on postoperative sagittal balance.

From Patient Reported Outcomes, we can see that the two surgical methods have achieved significant surgical effects, as well as the fact that the postoperative pain has been well relieved and the dysfunction of the cervical spine and thoracic and lumbar spines has also been significantly recovered, thus indicating that the two operations have fully removed the tumour and have an obvious effect on spinal canal decompression. Moreover, the ASIA grade was improved after the operation, and there was no significant difference between the two groups in terms of therapeutic efficacy.

There have been many studies on the loss of spinal ROM after screw fixation, especially for the fixation of more than three segments. The extension and flexion movements of the skull and neck mainly occur in the atlantooccipital joint, and the rotation movement mainly occurs in the atlantoaxial joint, accounting for half of the entire rotation movement. Flexion–extension radiographs of the cervical spine confirmed the biomechanical effects of extension and flexion. During flexion, the cervical spine was slightly kyphotic, the foramen magnum is almost parallel to the C7 vertebral body, and the cervical vertebra is excessively lordosis during extension. Neck extension is accomplished by the contraction of the hemispinalis muscle, multifidus muscle and longhead muscle and the relaxation of the hemispinalis muscle. The anterior flexion and left and right lateral flexion of the neck are mainly the function of the scalenus muscle. If both sides contract together, anterior flexion can occur. If only one side contracts, lateral flexion will occur. In this movement, the trapezius muscle plays an assisting role, and the scalenus muscle and sternocleidomastoid muscle work together to rotate the lower cervical spine. The coordinated contraction of the unilateral splenius, posterior scalenus and contralateral sternocleidomastoid muscle can cause rotation between atlantoaxial vertebrae. When the tumour is exposed, the posterior muscle tissue will be destroyed, which will affect the ROM of the spine. Lamina replacement has less damage to muscle tissue, and can reconstruct muscle in situ, which can damage the ROM of the spine as little as possible. From the perspective of biomechanics, the flexion and extension of the cervical spine can be independently performed, but its lateral flexion and rotation are interrelated and coexisting, and there is a coupling phenomenon; specifically, lateral flexion must be accompanied by rotation (and vice versa). A previous study showed that c4 ~ 5, c5 ~ 6 and c6 ~ 7 are larger than c2 ~ 3 in the normal cervical mobility, as well as the fact that c4 ~ 5, c5 ~ 6 and c6 ~ 7 are also larger in the contribution of each segment to the overall mobility [30]. However, in this study, there was no significant difference between the two groups in any of the directions of cervical spine movement after the operation. On the one hand, the cervical spine ROM we measured included head motion interference, and it was not measured on the radiograph; on the other hand, it may be because there are fewer cervical cases included in this study, which may lead to selective bias. Cervical tumour resection rarely involves the atlantooccipital and atlantoaxial joint, which retains more flexion extension and rotation of the cervical spine. The ROM in all directions in the screw fixation after laminectomy group of the thoracic and lumbar spines patients was significantly lower than that in the lamina replantation group. Screw fixation after laminectomy group seriously damaged the posterior muscle group, and the fixed segments lost their motor function, which could only be compensated for by the upper and lower segments. In addition, due to the heavy load on the thoracic and lumbar spines, and to maintain the stability of the spine, the spine had to reduce part of the ROM in exchange for balance to reduce the incidence of ASD. Replantation of the vertebral lamina can well preserve the ROM of the thoracic and lumbar spines and reduce muscle dissection during the operation. After the operation, the muscles are reconstructed in situ, which allows for the spine to retain both good stability and ROM.

Obviously, if we can compare the surgical effects of the same surgical segment and the same pathological type separately, this study would be more accurate and more convincing; however, it would be more difficult to collect cases that meet this condition. Our unit has not been able to collect numerous cases, which is also a limitation of this article.

## Conclusions

Lamina replantation can be used as splendid methods for the treatment of intraspinal tumour. Lamina replantation can reduce the operation time, as well as reduce the occurrence of postoperative cerebrospinal fluid leakage, iatrogenic spinal stenosis, posterior soft tissue adhesion and ASD. These complications are reduced in comparison with the other mode of management and better preserve the mobility of the spine.

## Data Availability

The data are not publicly available due the privacy of patients included but are available from the corresponding author on reasonable request for academic research purpose.

## Abbreviations

CT: Computed tomography
MRI: Magnetic resonance imaging
BMI: Body mass index
ASD: Adjacent segment degeneration
VAS: Visual analog scale
ODI: Oswestry Disability Index
NDI: Neck Disability Index
ROM: Range of motion

## References

[1] Vinken P, Bruyn G. Handbook of clinical neurology. North-Holland 1968.

[2] Bhimani AD, Denyer S, Esfahani DR, Zakrzewski J, Aguilar TM, Mehta AI. Surgical complications in intradural extramedullary spinal cord tumors—an ACS-NSQIP analysis of spinal cord level and malignancy. 10.1016/j.wneu.2018.06.014 29902605

[3] Miyakoshi N, Kudo D, Hongo M, Kasukawa Y, Shimada Y. Intradural extramedullary tumor in the stenotic cervical spine resected through open-door laminoplasty with hydroxyapatite spacers: Report of two cases. BMC Surg 2018;18(1). 10.1186/s12893-018-0372-9 PMC5996514 29890965

[4] Denis F (1983). The three column spine and its significance in the classification of acute thoracolumbar spinal injuries. Spine.

[5] Abeloos L, De Witte O, Riquet R, Tuna T, Mathieu N (2011). Long-term outcome of patients treated with spinal cord stimulation for therapeutically refractory failed back surgery syndrome: a retrospective study. Neurochirurgie.

[6] Radu AS. Failed back syndrome and epidural fibrosis;2000.

[7] Lee S, Cho Y, Kwon Y (2014). Neurological outcome after surgical treatment of intramedullary spinal cord tumors. Korean J Spine.

[8] Park Y, Kim S, Seo H (2019). Ligament-saving laminoplasty for intraspinal tumor excision: a technical note. World Neurosurg.

[9] Raimondi AJ, Gutierrez FA, Rocco CD (1976). Laminotomy and total reconstruction of the posterior spinal arch for spinal canal surgery in childhood. J Neurosurg.

[10] Song Z, Zhang Z, Ye Y, Zheng J, Wang F. Efficacy analysis of two surgical treatments for thoracic and lumbar intraspinal tumours. BMC Surg 2019;19(1). 10.1186/s12893-019-0602-9 PMC6734390 31500614

[11] Lee YS, Kim YB, Park SW (2015). Spinous process-splitting hemilaminoplasty for intradural and extradural lesions. J Korean Neurosurg Soc.

[12] Soto-Hernandez M, Garcia-Mateos R, Chavez RSM, Kite G (2010). Surgical removal of spinal mass lesions with open door laminoplasty. Cen Eur Neurosurg.

[13] Levin DA, Hale JJ, Bendo JA (2007). Adjacent segment degeneration following spinal fusion for degenerative disc disease. Bull NYU Hosp Jt Dis.

[14] Ueda H, Huang R, Lebl D (2015). Iatrogenic contributions to cervical adjacent segment pathology: review article. HSS J.

[15] Oral S, Tumturk A, Kucuk A, Menku A. Cervical hemilaminoplasty with miniplates in long segment intradural extramedullary ependymoma: case report and technical note. Turk Neurosurg 2018;28(1). 10.5137/1019-5149.JTN.14780-15.2 27593777

[16] Nakai O, Ookawa A, Yamaura I (1991). Long-term roentgenographic and functional changes in patients who were treated with wide fenestration for central lumbar stenosis. J Bone Jt Surg Am.

[17] Samartzis D, Gillis CC, Shih P, O'Toole JE, Fessler RG. Intramedullary spinal cord tumors: part ii-management options and outcomes. SAGE Publications 2016(2). 10.1055/s-0035-1550086 PMC4771497 26933620

[18] Asthagiri AR, Mehta GU, Butman JA, Baggenstos M, Lonser RR (2011). Long-term stability after multilevel cervical laminectomy for spinal cord tumor resection in von Hippel–Lindau disease. J Neurosurg Spine.

[19] Mcgirt MJ, L G-AG, Parker SL, Sciubba DM, Ali B, Jean-Paul W, Gokaslan ZL, George J, Witham TF. Short-term progressive spinal deformity following laminoplasty versus laminectomy for resection of intradural spinal tumors: analysis of 238 patients. Neurosurgery 9(10):89S. 10.1227/01.NEU.0000367721.73220.C9 20404708

[20] Byvaltsev V, Polkin R, Kalinin A, Kravtsov M, Belykh E, Shepelev V, Satardinova E, Manukovsky V, Riew K. Laminoplasty versus laminectomy in the treatment of primary spinal cord tumors in adult patients: a systematic review and meta-analysis of observational studies. Asian Spine J 2023. 10.31616/asj.2022.0184 PMC10300886 36717092

[21] Thakur NA. Laminoplasty: indication, techniques, and complications. Semin Spine Surg 2014;26(2):91–99.

[22] Larocca H, Macnab I. The laminectomy membrane. Studies in its evolution, characteristics, effects and prophylaxis in dogs. Bone Jt J 1974;56B(3):545–550. 4421702

[23] Winter RB, Hall JE (1978). Kyphosis in childhood and adolescence. Spine.

[24] Yasuoka S, Peterson HA, Maccarty CS (1982). Incidence of spinal column deformity after multilevel laminectomy in children and adults. J Neurosurg.

[25] Kapural L, Peterson E, Provenzano D, Staats P. Clinical evidence for spinal cord stimulation for failed back surgery syndrome (FBSS): systematic review. Spine 2017:S61–S66. 10.1097/BRS.0000000000002213 28441313

[26] Yun S, Kim D, Do H, Kim S (2017). Clinical insomnia and associated factors in failed back surgery syndrome: a retrospective cross-sectional study. Int J Med Sci.

[27] Liao Z, Fogel GR, Wei N, Gu H, Liu W (2015). Biomechanics of artificial disc replacements adjacent to a 2-level fusion in 4-level hybrid constructs: an in vitro investigation. Med Sci Monit Int Med J Exp Clin Res.

[28] Jaramillo JJ. Current reviews in musculoskeletal medicine Volume: 1 ISSN: 1935–973X ISO Abbreviation: Curr Rev Musculoskelet Med Publication Date: 2008 Jun. De La Torre.

[29] Luo J, Gong M, Huang S, Yu T, Zou X (2015). Incidence of adjacent segment degeneration in cervical disc arthroplasty versus anterior cervical decompression and fusion meta-analysis of prospective studies. Arch Orthop Trauma Surg.

[30] Muhle C, Wiskirchen J, Weinert D, Falliner A, Wesner F, Brinkmann G, Heller M (1998). Biomechanical aspects of the subarachnoid space and cervical cord in healthy individuals examined with kinematic magnetic resonance imaging. Spine.

